# Primary Hyperparathyroidism in a Post-total Thyroidectomy Patient: An Unexpected Diagnosis

**DOI:** 10.7759/cureus.33308

**Published:** 2023-01-03

**Authors:** Ana F. Batista, Sara Fontaínhas, Sónia C. Pereira

**Affiliations:** 1 Serviço de Medicina Interna, Hospital Distrital da Figueira da Foz, Figueira da Foz, PRT

**Keywords:** chronic kidney disease (ckd), case-report, total thyrodectomy, parathyroid adenoma, primary hyperparathyroidism, hypercalcemia

## Abstract

Hypercalcemia is commonly encountered in the clinical setting. Although primary hyperparathyroidism resulting from a single parathyroid adenoma is the most common cause, in patients who undergo total thyroidectomy, especially when there is no history of radiation exposure nor parathyroid autotransplantation, it becomes an even more unexpected diagnosis. Because the majority of patients are asymptomatic, the diagnosis often is made incidentally. However, with long-standing disease, as parathyroid hormone and blood calcium levels rise, symptoms become more noticeable, and its clinical manifestations can affect nearly every organ system in the body.

We present the case of a 79-year-old woman with a history of surgical hypothyroidism secondary to total thyroidectomy, hypertension and chronic kidney disease, who was admitted to the Emergency Department after an episode of syncope. She mentioned abdominal pain and vomiting in the previous week and paresthesia of both hands and feet over the last months. The initial testing identified a first-degree auriculoventricular block, a worsened renal function and severe hypercalcemia caused by primary hyperparathyroidism. The mainstay of treatment was aggressive fluid therapy, intravenous bisphosphonate and calcimimetic. Definitive treatment was achieved by the surgical removal of a mass located in the left thyroidectomy bed, compatible with a parathyroid adenoma. No further therapy was needed, as calcium levels gradually returned to normal.

## Introduction

Hypercalcemia is an important electrolyte disturbance, defined by a serum calcium concentration >10.5 mg/dL and, depending on calcium values, it is classified into mild, moderate and severe [1]. Primary hyperparathyroidism (PHPT), which is the leading cause of hypercalcemia, is the most common parathyroid disorder and solitary adenomas are responsible for more than 80% of all cases [1-5]. Other clinical conditions, such as medications, chronic kidney disease, granulomatous diseases and some types of cancer may additionally contribute to the hypercalcemic state [1]. The majority of patients with hypercalcemia are asymptomatic and therefore diagnosed during routine biochemical tests [1,2]. Whenever present, symptom frequency and severity correlate with calcium levels and mass weight so that calcium levels >14mg/dL, malignancy (also associated with high calcium levels) or giant adenomas (defined by weight >3.5g or diameter >2cm), can trigger life-threatening hypercalcemic crises [3-6]. Severe manifestations can affect the neurological, muscular, gastrointestinal, renal and cardiovascular systems [1,2].

In the specific case of previously thyroidectomized patients, severe hypercalcemia secondary to PHPT is a rare diagnosis, even more, when there is no history of cervical irradiation procedure nor parathyroid autotransplantation. Because it represents a potentially life-threatening condition, high suspicion is crucial to achieve a timely diagnosis, start early treatment and prevent consequences [2].

## Case presentation

The authors present the case of a 79-year-old woman with a history of hypertension, chronic kidney disease (kidney disease improving global outcomes (KDIGO) classification G3bA1), surgical hypothyroidism (underwent total thyroidectomy 15 years before for non-toxic multinodular goiter), medicated with an angiotensin receptor antagonist, thiazide diuretic, loop diuretic, and levothyroxine. She went to the Emergency Department due to a first, 30 seconds lasting, and self-limited episode of syncope at rest, while sitting, with prodromal symptoms of dizziness, sweating and pallor, without associated trauma. There were also complaints of abdominal pain from the previous week, with two episodes of postprandial vomiting on the day before admission and a tendency to constipation. She also mentioned paresthesia in all fingers and toes over the last months, which was progressively worsening. She denied chest pain, palpitations, headache, dyspnea, urinary complaints, fever, or other B symptoms.

**Figure 1:**
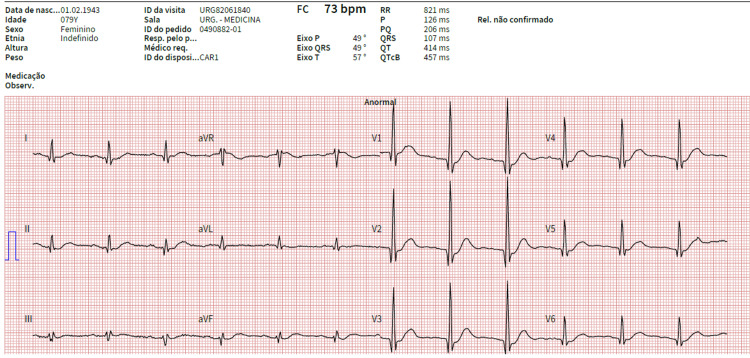
Electrocardiogram with first-degree AV block, prominent R-waves in V1-V3 with associated Q-waves and HR of 73 bpm.

On examination, she was conscious, oriented in time and space, without amnesia, and cooperating. There were no signs of trauma or seizure stigmas. The paresthesias mentioned in the clinical history were confirmed, with negative Tinel and Phalen maneuvers (provocative tests used in the diagnosis of carpal tunnel syndrome). No speech, visual, or motor alterations were observed. The evaluation of vital signs and blood glucose showed values ​​within normal limits. Clinical examination was normal apart from mild diffuse abdominal discomfort on palpation. Peripheral edema was not evident. The electrocardiogram showed first-degree atrioventricular block, prominent R-waves from V1 to V3 with Q waves (suggestive of posterior fibrosis or septal ventricular hypertrophy), and a heart rate (HR) of 73bpm (Figure 1).

The blood testing revealed normochromic normocytic anemia of 10.7g/dL (N 12.5-16.0g/dL) and a worsened renal function, with urea of 54.2mg/dL (N 8-23mg/dL) and creatinine level of 2.1mg/dL - baseline value of 1.6mg/dL (N 0.5-0.9mg/dL), in addition to multiple electrolyte disorders - hyponatremia, hypokalemia, hypochloremia, hypophosphatemia, with hypercalcemia being the most relevant of them all, with a corrected calcium of 16.5mg/dL (N 8.4-9.7mg/dL), along with a very high PTH value of 1240 pg/mL (N 15-65pg/mL). There was also a vitamin D deficit. Inflammatory markers were within normal limits and the measurement of thyroid hormones showed no alterations (Table 1).

**Table 1:** Laboratory evaluation

Test	Result	Reference range
Hemoglobin	10.7 g/dL	12.5-16.0 g/dL
White blood cells	9.89 x10^3/uI	4.0-10.5 x10^3/uI
Platelets	212 x10^3/µL	150-450 x10^3/µL
Urea	54.2 mg/dL	8-23 mg/dL
Creatinine	2.1 mg/dL	0.5-0.9 mg/dL
Sodium	132 mEq/L	136-145 mEq/L
Potassium	3.3 mEq/L	3.5-5.1 mEq/L
Chloride	93 mEq/L	96-107 mEq/L
Corrected calcium	16.5 mg/dL	8.4-9.7 mg/dL
Phosphorus	2.5 mg/dL	2.7-4.5 mg/dL
Magnesium	1.7 mEq/L	1.4-2.1 mEq/L
Vitamine D	9.6 ng/mL	>20 ng/mL
PTH	1240 pg/mL	15-65 pg/mL
TSH	0.87 µUI/mL	0.27-4.2 µUI/mL
T4L	1.67 ng/dL	0.92-1.68 ng/dL
Amylase	107 U/L	28-100 U/L
Lypase	43.0 U/L	13-60 U/L
C-Reactive protein	<0.60 mg/dL	<5 mg/dL
Troponin	52 pg/mL	<50 pg/mL
D-dimers	284 ng/mL	<243 ng/mL

In view of these changes, vigorous fluid therapy was initiated (normal saline, 3L intravenously daily for three days, then tapered) with ionic and vitamin D supplementation, according to needs, loop diuretic and zoledronic acid (bisphosphonate). On the other hand, drugs with hypercalcemic potential or harmful to the kidney were stopped. At a later stage, cinacalcet (calcimimetic) was also started. The repeat calcium levels after one week were 12.3mg/dL.

**Figure 2:**
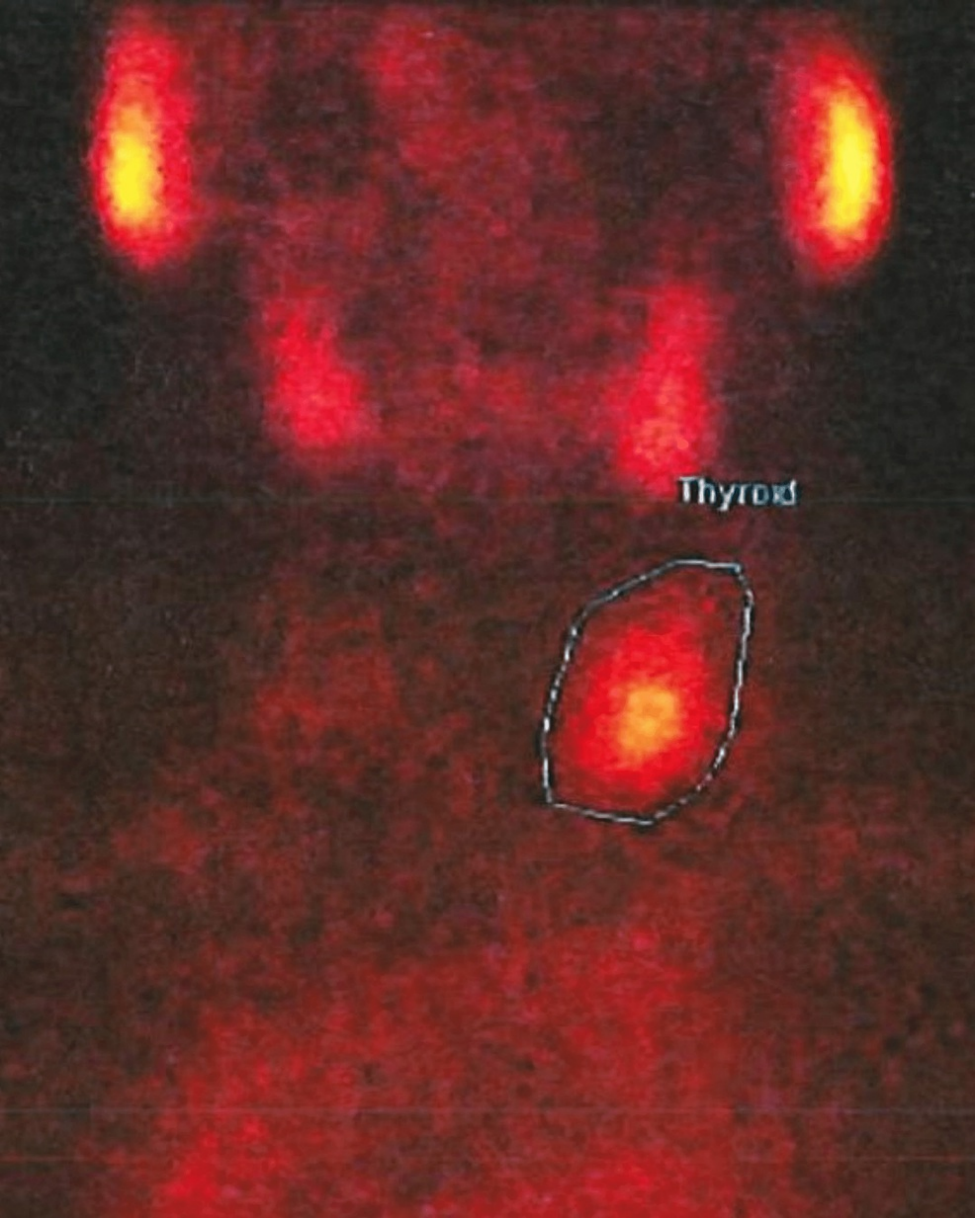
99m Tc-Sestamibi scintigraphy. The area radiotraced is highlighted, compatible with parathyroid tissue.

The subsequent study included abdominal and renal ultrasounds that showed kidneys with loss of corticomedullary differentiation, without other relevant alterations. Cervical ultrasound revealed a hypoechogenic lesion, with regular and well-defined contours, dimensioned 42x24x32mm, located in the left thyroidectomy bed, which did not deviate from the air column or the left jugular-carotid axis. No cervical adenopathies or right thyroid remnants were identified. 99mTc-Sestamibi scintigraphy showed an area of tracer uptake, compatible with a large formation of the left parathyroid gland (Figure 2).

**Figure 3:**
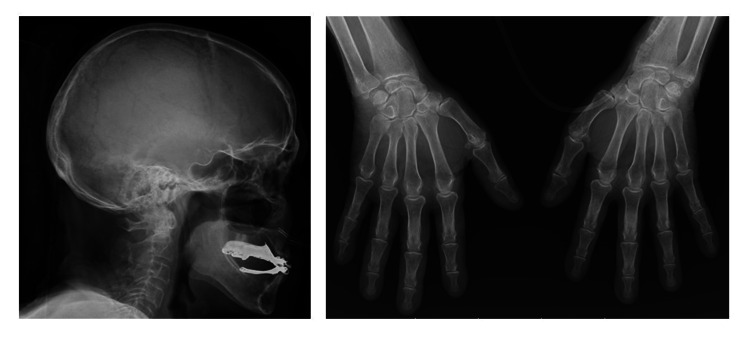
X-ray of the skull (left) and hands (right), without alterations.

X-rays of the skull and hands were also performed, and both showed no alterations (Figure 3).

The patient was hospitalized, under continuous electrocardiographic monitoring, with no evidence of arrhythmia. Clinical and analytical stabilization was gradually achieved over the course of two weeks, after which she underwent left parathyroidectomy (pre-surgical calcium 9.8 mg/dL), with pre and 10-minute post-procedure PTH measurements of 1,009 and 210.5 pg/mL, respectively. At the time of discharge, in addition to being clinically asymptomatic, the analytical control showed calcium of 9.3 mg/dL and PTH of 6.7 pg/mL. The patient maintains regular follow-ups. The anatomopathological evaluation revealed a parathyroid adenoma.

## Discussion

Hypercalcemia is defined by a serum calcium concentration >10.5 mg/dL and is classified into mild, moderate, and severe, whether calcium levels are 10.5-12 mg/dL, 12.1-14 mg/dL or >14 mg/dL, respectively [1]. A thorough history and physical examination should be performed, in order to identify signs and symptoms of organ dysfunction, as well as medications that may alter calcium homeostasis. Past medical history of previous or current malignancies, head/neck irradiation as well as family history of hyperparathyroidism should be questioned [1,7].

Although it has multiple etiologies, 90% of all cases of hypercalcemia result from PHPT or malignancy. Therefore, the most important analysis during its investigation is the measurement of PTH [1-3,7]. The group of etiologies mediated by an excess of PTH (PTH-dependent hypercalcemia), of which this clinical case is an example, includes adenoma/hyperplasia of the gland, familial hypocalciuric hypercalcemia and multiple endocrine neoplasias (MEN) syndromes [8]. Less frequently, parathyroid carcinoma should be considered when serum calcium levels exceed 14mg/dL and are associated with a palpable cervical mass [3]. The other group is that of malignant causes, mediated by the action of PTHrp (PTH - related peptides), where renal carcinoma, leukemias, or lymphomas may be associated with increased calcium levels (PTH-independent hypercalcemia) [8]. In this case, and similarly to what is often seen for other disorders, the etiology may be multifactorial, contributing concomitantly to the state of hypercalcemia, advanced chronic kidney disease (related to tertiary hyperparathyroidism), the effect of thiazide diuretics (increase calcium reabsorption) and vitamin D deficiency (results in compensatory mild PTH elevation) [4,7,9].

From a clinical point of view, most patients are asymptomatic [1,2]. Higher calcium levels or hypercalcemia of acute onset are associated with more frequent and severe signs and symptoms that include multisystemic manifestations, according to the mnemonic “stones, bones, abdominal moans and psychic groans”, which refers to renal, skeletal, gastrointestinal and neuromuscular involvement. Psychiatric and cardiovascular manifestations may also occur and calcium values >15 mg/dL are even associated with an increased risk of cardiac arrest [1,7]. In this case, there were renal alterations due to dehydration (worsening of creatinine compared to baseline values, with increased nitrogen retention); gastrointestinal symptoms, such as abdominal pain, nausea, vomiting, and constipation; neuromuscular, classified as a sensory, symmetrical and distal polyneuropathy (which denotes chronicity) and cardiovascular manifestations, as the electrocardiogram showed prolongation of the PR segment - The syncope episode may have resulted from the synergism of volume depletion and the potential arrhythmogenic effect associated with hypercalcemia or simply represent a case of autonomic dysfunction, attributable to normal aging process [3,4,7,9,10]. The skeletal manifestations, characterized by the typical radiological characteristics of salt and pepper seen in the skull, and periosteal reabsorption of the distal phalanges, were not observed (Figure 3) [4].

The diagnosis of a parathyroid mass was suspected by the analytical finding of PHPT (hypercalcemia and elevated PTH) and later confirmed by ultrasound (non-invasive and low-cost procedure) and 99mTc-Sestamibi scintigraphy (sensitivity of 90% in detecting parathyroid adenomas) [5,9].

The therapeutic approach to hypercalcemia due to PHPT, in the acute setting, depends more on the presence of symptoms, rather than on the serum calcium level or underlying etiology, and includes both medical and surgical strategies [4,6]. The mainstay of treatment is aggressive fluid therapy, which acts by reversing intravascular volume contraction, promotes renal calcium excretion, and should ensure a diuresis of 100-150 mL/h (although the choice of fluid must be individualized according to electrolytes, 0.9% saline is the most used) and intravenous bisphosphonate, that exerts anti-osteoclastic activity and reduces calcium levels within 24-72 hours, with a permanent effect for 2-4 weeks (the two options approved are pamidronate and zoledronic acid). Furosemide, glucocorticoids, calcitonin, and dialysis are indicated in some patients [1,4,6].

Surgical intervention constitutes the only form of definitive treatment and is reserved for cases of clinically symptomatic PHPT, calcium 4.5 mg/dL above the upper limit of normal, creatinine clearance <60 mg/dL, low bone mineral density, history of frailty fracture or age <50 years [4,6,7]. As there is a high risk of cardiac complications secondary to the sudden decrease in calcium levels, surgery should be postponed until a safe serum calcium level is reached, in the absence of symptoms directly attributable to the hypercalcemic state [6]. In this case, the surgical approach was only possible after two weeks of optimized therapy. Intraoperative PTH measurement helps to assess the success of the surgery, which is determined by a drop in the PTH value of >50% from baseline, 10 minutes after excision (Miami Criteria) - in this case, the preoperative PTH values and at 10 minutes after the adenoma excision were 1009 and 210.5pg/mL, respectively, which attests to surgical success [4,5].

PHPT, which was the main responsible for this patient's severe hypercalcemia, is the most common parathyroid disorder and constitutes the third most prevalent endocrine disease [3,4]. It can occur at any age, although most appear between the ages of 50-60, affects significantly more females than males and a solitary adenoma is responsible for more than 80% of all cases [2,4,5]. In thyroidectomized patients, it is usually associated with a history of cervical irradiation procedures or parathyroid autotransplantation, none of which were present in this patient background. Symptom severity correlates with calcium levels and mass weight so that, although the most likely scenario is of an incidental diagnosis, malignancy or giant adenomas (defined by weight >3.5g or diameter >2cm), can trigger life-threatening hypercalcemic crisis [3-6]. The parathyroid crisis is rare and is characterized by extremely high levels of PTH, associated with severe hypercalcemia of acute onset, triggering multiorgan dysfunction (metabolic encephalopathy, renal failure, gastrointestinal symptoms, and cardiac arrhythmia) [6].

## Conclusions

Patients usually seek medical consultation upon symptoms that are alarming, disregarding other potentially important complaints. In this case, although there was a long-standing history of paresthesias and more recently gastrointestinal symptoms, only when syncope occurred the patient went to the emergency department, which allowed for the diagnosis and treatment of severe hypercalcemia, in the setting of PHPT.

Both hypercalcemia and PHPT represent a major challenge, as not only are most patients asymptomatic, but when symptoms do occur, most are nonspecific, which translates into a delayed diagnosis, leaving open the possibility of numerous severe consequences. A high degree of clinical suspicion is required to promptly diagnose these patients, particularly in emergency room scenarios.
